# Displacements Study of an Earth Fill Dam Based on High Precision Geodetic Monitoring and Numerical Modeling

**DOI:** 10.3390/s18051369

**Published:** 2018-04-27

**Authors:** Luis Enrique Acosta, M. Clara de Lacy, M. Isabel Ramos, Juan Pedro Cano, Antonio Manuel Herrera, Manuel Avilés, Antonio José Gil

**Affiliations:** 1 Departamento de Construcciones, Universidad de Holguín, Holguín 80100, Cuba; luis.acosta@uho.edu.cu; 2 Departamento de Ingeniería Cartográfica, Geodesia y Fotogrametría, Universidad de Jaén, Jaen 23071, Spain; miramos@ujaen.es (M.I.R.); aherreraolmo@gmail.com (A.M.H.); maviles@ujaen.es (M.A.); ajgil@ujaen.es (A.J.G.); 3 CEACTierra—Centro de Estudios Avanzados de Ciencias de la Tierra, Jaen 23071, Spain; 4 4D Geoservices, Jaen 23008, Spain; jpcc0001@red.ujaen.es

**Keywords:** high-precision leveling, GNSS, Finite Element Method, settlements, horizontal displacements

## Abstract

The aim of this paper is to study the behavior of an earth fill dam, analyzing the deformations determined by high precision geodetic techniques and those obtained by the Finite Element Method (FEM). A large number of control points were established around the area of the dam, and the measurements of their displacements took place during several periods. In this study, high-precision leveling and GNSS (Global Navigation Satellite System) techniques were used to monitor vertical and horizontal displacements respectively. Seven surveys were carried out: February and July 2008, March and July 2013, August 2014, September 2015 and September 2016. Deformations were predicted, taking into account the general characteristics of an earth fill dam. A comparative evaluation of the results derived from predicted (FEM) and observed deformations shows the differences on average being 20 cm for vertical displacements, and 6 cm for horizontal displacements at the crest. These differences are probably due to the simplifications assumed during the FEM modeling process: critical sections are considered homogeneous along their longitude, and the properties of the materials were established according to the general characteristics of an earth fill dam. These characteristics were taken from the normative and similar studies in the country. This could also be due to the geodetic control points being anchored in the superficial layer of the slope when the construction of the dam was finished.

## 1. Introduction

Dams which are large and important engineering structures storing millions of cubic meters of water have to accommodate substantial horizontal and vertical movements due to the presence of various external and internal loads. When these movements, also called deformations and displacements, reach certain critical limits, catastrophic damage may occur, even leading to the total collapse of the dam. As a result, it becomes a necessity to monitor such movements at certain intervals because of their significant social, technical, and economical impact [1]. In general, dams are systematically surveyed on the basis of geodetic and geotechnical methods, but presentations of results are difficult to find in the literature due to their confidential nature. Several instruments and procedures have been developed for monitoring and predicting dam behavior. Most of them are based on geodetic techniques. For example some authors have used GNSS techniques [2,3]; in other cases, monitoring is based on InSAR (Interferometric Synthetic Aperture Radar) methods [4,5,6]; spirit leveling is the most commonly-used technique to study the settlements in civil engineering [7,8,9], and motorized control total stations constitute a modern method for monitoring deformations [10]. It can be seen in [11] that the most precise monitoring surveys do not fully serve their purpose if they are not properly evaluated and used in a global integrated analysis as a cooperative interdisciplinary effort. Therefore, the combination of monitoring and numerical modeling of deformations is essential for studying the processes occurring in engineering structures and in rock masses at the construction and post-construction stages [12]. The approach is based on a combination (integration) of deterministic modeling (prediction) of deformations, with the monitoring results obtained from geodetic and/or geotechnical measurements of displacements and deformations of the object investigated. Examples of monitoring structural deformations based on this approach can be seen in [13]. In this case the deformation monitoring of the Mornos dam (Greece) is based on precise leveling data of a large number of control stations established along the crest and the inspection gallery of the dam. Settlements are derived from magnetic extensometers placed inside the dam body. Following a similar approach, ref. [14] evaluated the dynamic behavior of the Ermenek Dam, based on conventional geodetic measurements and Finite Element Method (FEM) analyses obtained during the first filling period. In both cases, the results show good agreement between geodetic and numerical methods. 

In this paper, we study the behavior of an earth fill dam, analyzing deformations using high precision geodetic techniques, and those obtained with FEM. In particular, settlements were estimated from high-precision leveling observations [15], and horizontal displacements were estimated from GNSS measurements. In Section 2, a description of the dam is summarized. The high-precision geodetic measurements (high precision leveling and GNSS measurements) are explained in Section 3. In Section 4, the basic information on the finite element modeling of the structure is introduced. Finally, the authors compare the deformations determined by geometric and numerical modeling.

## 2. The Arenoso Reservoir

**Figure 1:**
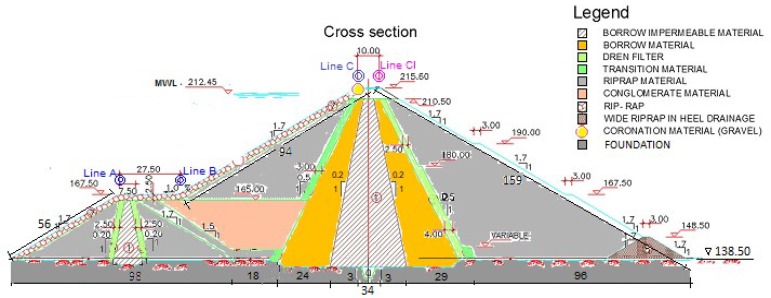
Cross-section of the Arenoso dam. Lines A and B represent the berms; lines C and CI represent the downstream and upstream crest, respectively [15]. Units in meters.

The Arenoso reservoir is an embankment dam, with a central clay core, slates and greywacke shoulders. The core is covered downstream by a filter material, and upstream by a transition material. It also has a cofferdam with a similar section and materials, which remains leaning against the dam’s body. The dimensions of the dam are 80 m high and 1480 m long at its crest, and in its construction more than 3 million m^3^ of materials were used, creating a waterproof barrier able to hold 160 million m^3^ as a useful reservoir [16]. A perimeter gallery was constructed in the dam’s foundation for observation purposes (Figure 1). The construction of the dam began in April 2004 and finished in November 2006. The first control of settlements was carried out in February, March, April and July 2007 after the first filling of the dam in November 2006. An optical level NIKON AC-2SG was used for the leveling tasks. The results of the leveling led to displacements of up to 11 cm in 6 months. Monitoring with high precision geodetic techniques began in 2008. Up to now, seven high precision geodetic surveys have been carried out. Unfortunately, no geodetic data are available between 2009 and 2012.

## 3. The High Precision Geodetic Monitoring System

**Figure 2:**
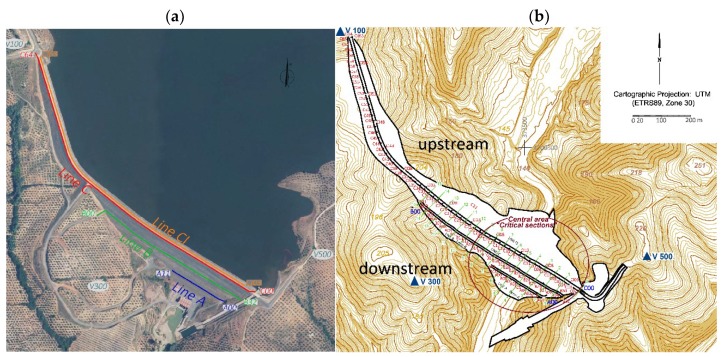
(**a**) Locations of control lines at the berms (lines A and B) and on the downstream (line C) and upstream side of the crest (line CI). (**b**) Locations of the control points for high precision geodetic observations and distribution of the cross sections (critical sections) for numerical modeling.

The methodology of the geodetic monitoring of the dam is based on the control of possible displacements of its crest and principal axis. The methodology of this analysis is based on monitoring of the position of several points at different periods. With this objective, four monitoring lines corresponding to the main axes of the dam were established. They correspond to the berms (lines A and B) and the downstream and upstream crest (lines C and CI) respectively. They are shown in Figure 2a.

### 3.1. Settlement Monitoring

**Figure 3:**
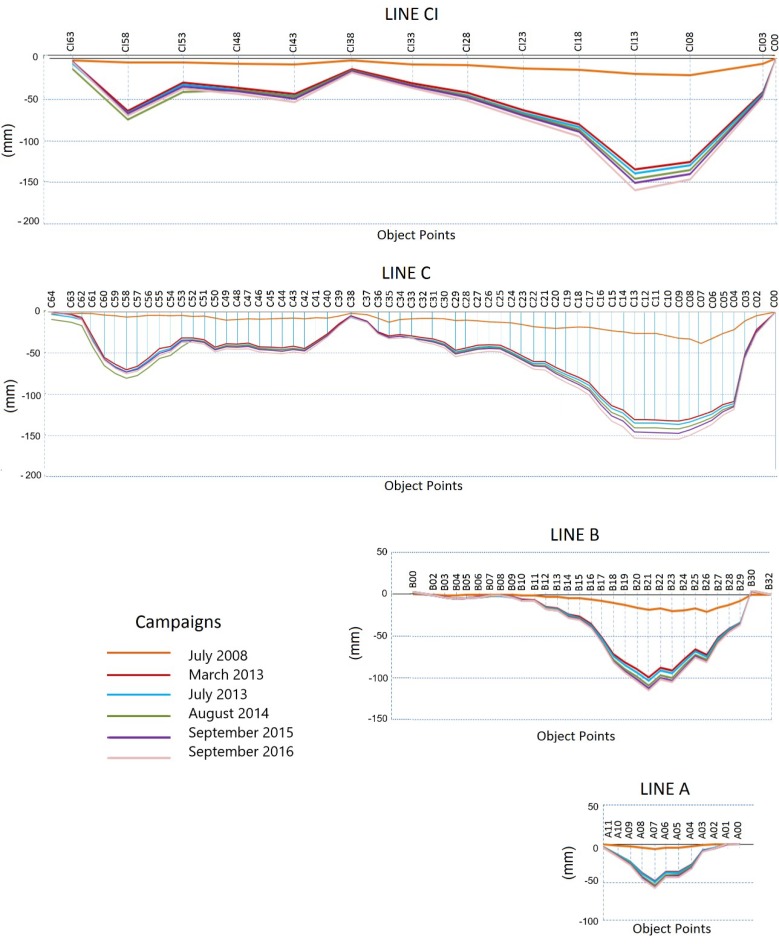
Accumulative displacements estimated by high precision leveling in lines A, B, C and CI in mm. Period 2008–2016, [15].

Seven high-precision leveling surveys were carried out twice per year in 2008 and 2013, and once in August 2014, September 2015 and September 2016. Relative vertical displacements of the control stations of the dam were measured using high-precision leveling (LEICA DNA03 digital level and 3-metreInvar bar-coded staffs), with respect to fixed benchmarks placed at the main axes of the dam, in particular A00, B00 and C00. These points are used to realize the reference frame at every line. Relative elevation changes for each benchmark and each survey were measured. These values were used to estimate the accumulative displacements of the dam from 2008. The results can be seen in Figure 3. These results indicate downstream motion of the thrust block center of the dam during the fall and winter. The settlement reaches the maximum here, with a value of −16 cm in 2016 with respect to February 2008. The displacements observed at the berms of the dam exhibit a similar trend to those observed at the crest, but they are significantly smaller, as expected. The accumulative vertical displacements indicate that the magnitude of the movements decreases in time, confirming the idea that the dam tends to stabilize.

### 3.2. Horizontal Displacements

Seven GNSS surveys were carried out to monitor horizontal deformations. Efforts were made to take measurements twice per year, while reservoir level fluctuations and rainfall height data were almost equally-spaced; however, this was finally impossible due to economic reasons. For the same reasons, no geodetic data are available from 2009 to 2012. In fact, the measurements were taken in correspondence with leveling observations (i.e., twice per year in 2008 and 2013, and once in August 2014, September 2015 and September 2016). In 2008, the equipment consisted of six dual frequency Leica GX 1200 receivers with AX1202 antennas (Leica Geosystems AG, Heerbrugg, Switzerland). The observation sessions lasted five days, with the following characteristics: static observations with a sampling rate of 5 s and a cut off angle equal to 10°. Three receivers were placed at the three external pillars (V100, V300 and V500), and the other three moved along some selected control points at the berms and crest. The duration of the sessions was 24 h at the external pillars and two hours at the other points. The selected control points were measured at least twice, in order to study their repeatability. Since 2013, we have had the use of 13 Leica GR10 receivers with AR10 antennas (Leica Geosystems AG, Heerbrugg, Switzerland). The surveys lasted 8 days in a static mode, with a 30-s sampling rate, and a cut off angle equal to 10°. Three receivers were placed at the external pillars, and the other 10 moved along all the control points. The sessions lasted 24 h for the external pillars, and 12 h for the control points. The control points were measured twice in order to study their repeatability. It is important to note that V100, V300 and V500 are classical pillars in the field with forced centering system. The points of the control network are materialized from leveling nails along the four main lines A, B, C and CI. These nails are protected by a hard plastic case, in order to prevent deterioration due to weather conditions. They are embedded 40 cm into the ground, and at the base of the casing is a layer of concrete for stability and support. GPS surveys were carried out with tripods with fixed-length legs at the control points and optical plumb. 

**Figure 4:**
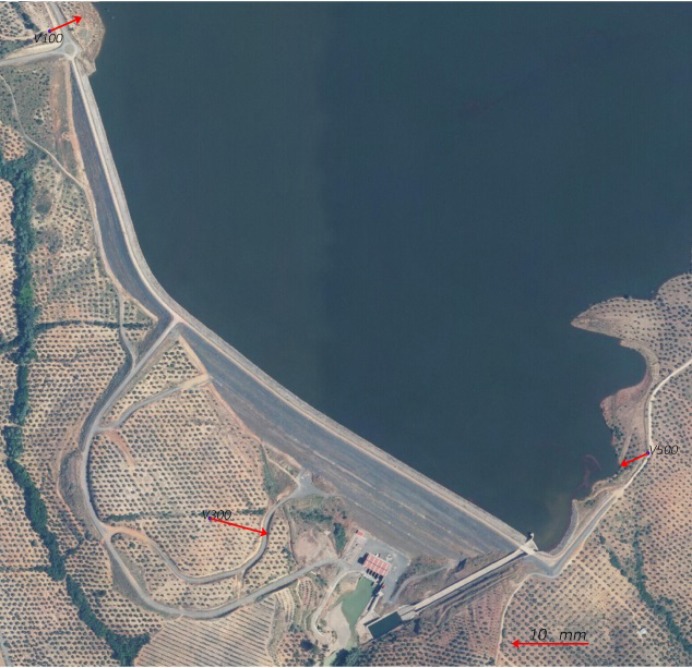
Horizontal displacements estimated at the external pillars V100, V300 and V500.

In 2008, due to the instruments and economic reasons, the surveyed control points were the following: A02, A05, A07 in line A; B00, B07, B15, B17, B22 and B27 in line B; C00, C04, C07, C10, C12, C15, C19, C23, C26, C28, C30, C33, C37, C41, C45, C53, C58 and C64 in line C; CI03, CI08, CI13, CI18, CI23, CI28, CI33, CI38, CI43, CI48, CI53 and CI63 in line CI. Since 2013, all control points have been surveyed. So, data from the 2008 and 2013 surveys cannot be considered to be completely homogeneous and of the same accuracy. Therefore, the horizontal displacements study is divided into two periods: one from 2008 to 2013, and the other one from 2013 to 2016. Data belonging to the first period were processed using Leica Geo Office Software (Leica Geo Office Software Version 7.0) [17], with the following options: phase double difference observations, cut off angle equal to 10°, precise ephemeris, iono-free with fixed ambiguities solution and Saastamoinen model to reduce the tropospheric effect. First, ITRF2005 (19/02/2008) coordinates of the pillar V100 were estimated using the observations from the IGS (International GNSS Service) stations of San Fernando (SFER) and Villafranca (VILL) in Spain, Cagliari (CAGL) in Italy and Rabat (RABT) in Morocco. The maximum horizontal repeatability of the coordinates reaches one mm, but in general, is below that. After that, the V100 coordinates were fixed to estimate the positions of the V300 and V500 stations. In this case, the planimetric repeatability of the V300 and V500 coordinates is better than 1 mm. For the second survey, we fixed the V100 point, and we estimate the coordinates of the V300 and V500 stations. The horizontal repeatability of these solutions is again better than 1 mm, but the differences between the V300 and V500 coordinates estimated from the February and July 2008 surveys reach 7 mm. Point C00 is on a reinforced concrete wall, and therefore should be stable. In order to analyze the stability of V100, V300 and V500, ITRF2005 (19/02/2008), coordinates of the point C00 were estimated using the stations of SFER, VILL, CAGL and RABT. Subsequently, C00 was fixed, and V100, V300 and V500 were estimated using the data from February and July 2008 surveys. The repeatability of the estimated coordinates is always better than 1 mm. The corresponding displacements are shown in Table 1 and Figure 4. The orientation of these displacement vectors is a downward slope, so the possibility of a rotrotranslation between the February 2008 and July 2008 surveys is discarded. The results obtained suggest the external pillars could be less stable than expected.

The fact that the point C00 is placed on a reinforced concrete wall increases its stability. Therefore, the adapted methodology is to define the reference frame using the stations of SFER, VILL, CAGL and RABT to estimate C00 coordinates in ITRF2005 (19/02/2008). After that, the coordinates of C00 are fixed and V100, V300 and V500 coordinates are estimated for every survey. Finally, the coordinates of all the control points were estimated from V100, V300 and V500 for every survey.

**Figure 5:**
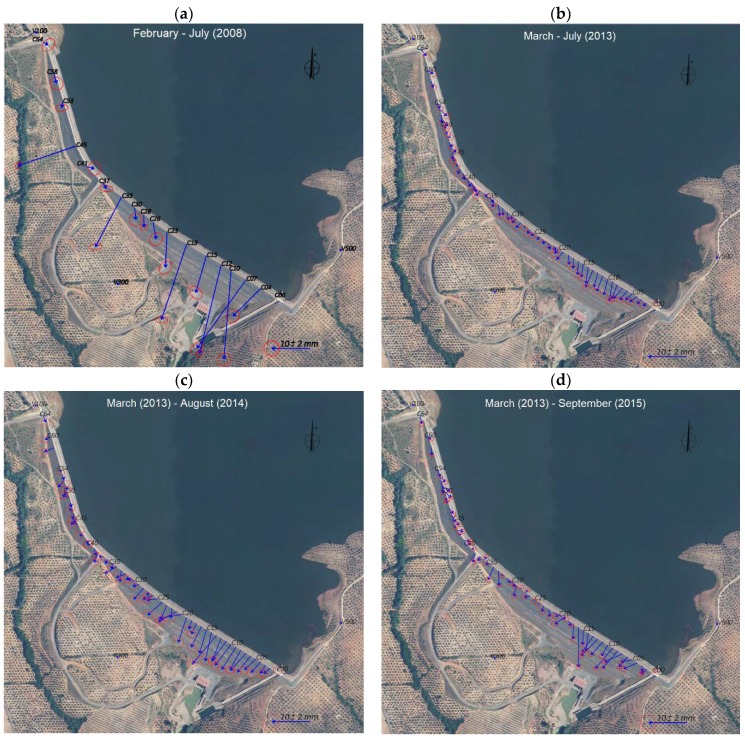

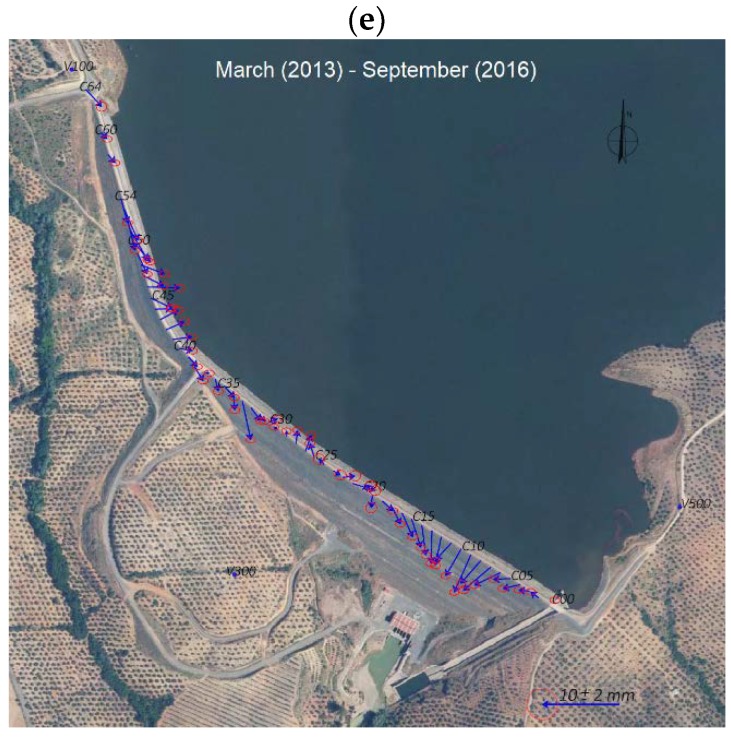
Horizontal displacements estimated by GPS in mm with error ellipses at 99% confidence. (**a**) Period February–July 2008; (**b**) period March–July 2013; (**c**) period March 2013–August 2014; (**d**) period March 2013–September 2015; (**e**) period March 2013–September 2016.

GNSS data from 2013 to 2016 were processed with Leica Geo Office Software (Leica Geo Office Software Version 7.0) [17], using the above options: phase double difference observations, cut off angle equal to 10°, precise ephemeris, iono-free with fixed ambiguities solution and Saastamoinen model to reduce the tropospheric effect. From 2013 to 2016, the horizontal displacements were computed with respect to the survey carried out in March 2013, since all these observations are completely homogeneous and of the same accuracy. Figure 5 shows the horizontal displacements estimated in line C (downstream crest) in time. All control points surveyed in 2008 are represented in Figure 5a. All control points surveyed in 2013, 2014, 2015 and 2016 are shown in Figure 5b–e, respectively. The name of the point is written only every five points, in order to make the figure clearer. Table 2 summarizes the statistics of the semimajor and semiminor axes of the error ellipses.

The results obtained from these two periods indicate the following facts: the area where the horizontal displacements are largest coincides with the area where the settlements reach their maximum (called critical area in Figure 2b); the trend of these displacements is downstream as a result of the water load; the maximum horizontal displacement is about 4 cm in 2008, afterwards the horizontal displacements decrease by values of up to a few mm; in 2008, large values are reached at points C45, C33, C23, C19, C12, C10 and C07. These points correspond to points in which inclinometric tubes were installed during the construction of the dam. To avoid any damage during the forced compaction, less energy was used. It is known that the precision of the coordinates estimated by GPS is, in general, overestimated. This is the reason why error ellipses are very small, so it is difficult to consider the displacements as significant. Authors such as Cocard et al. [18] say that a “realistic” error in GPS displacements is 15 times the statistical one. Some points, such as C25, C45 and C50 for example, show a changing behavior in time. It is important to note that the magnitude of the displacement is very small. That means that they are not significant against observed errors and systematic effects due to the short observation periods. The results obtained at the berms exhibit a similar trend to the displacements observed at the crest, but their magnitude is smaller.

## 4. Numerical Modeling

The SIGMA/W is a powerful Finite Element Method tool that can be used to model a wide range of stress-strain problems, in order to compute stress-deformation with or without the changes in pore-water pressures that arise from stress state changes. In addition, it is possible to model soil structure interaction using beam or bar elements. The most common application of the SIGMA/W is to compute deformations caused by earthworks such as foundations, embankments, excavations and tunnels [19].

The deformation of the foundation of the dam was estimated using two-dimensional linear elastic finite-element modeling techniques, performed using the SIGMA/W module of the GeoStudio 8 software package. Different stages of construction were incorporated, including the placement of fill dam and the filling of the reservoir. In this model, we considered the constituent models of materials, the interfaces between them, and the border conditions specific to the modeling.

The numerical modeling was based on the method of the finite elements for static deformation analysis. Sixteen sections were projected along the dam assuming homogeneity in the layers of the soils of the geological profiles. Furthermore, the areas with the maximum deformation were known from geodetic results (Figure 3 and Figure 5). They correspond to sectors 0 to 9 in Figure 2b. They are called “critical” sections. This central area will be the only one considered in our analysis.

### 4.1. Geotechnical Features

For the construction and configuration of the model, it is necessary to define the typical characteristics of one section placed in the central area of the dam. Table 3 summarizes the material properties used in this analysis. These were established according to Figure 1. In particular, the modeling by FEM was configured assuming the following simplifications: critical sections were considered homogeneous along their longitude and the properties of the materials were established from the normative and similar studies in the country, [20]. 

### 4.2. Results of the Numerical Modeling

**Figure 6:**
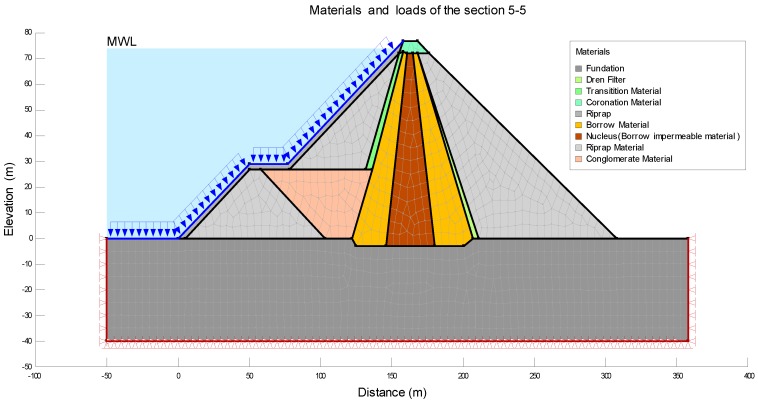
Detail of Section 5-5: considered layers with their materials and loads.

The modeling was carried out for the load of the Maximum Water Level (MWL) during the periods of construction, filling and early operational life. In order to estimate the settlements in the operational period it was necessary to combine the options Seep/W and Sigma/W, to carry out the analysis Load/deformation, and then to correlate with the tool Coupled Stress/PWP, being able to model the displacements in function of time, including the process of consolidation of the soil. As mentioned above, the central area (critical section) was the only one to be analyzed. In particular, section 5-5 was studied. Figure 6 was obtained from the configuration of Keyln and Draw tools. This figure shows the different layers with the corresponding material and loads.

**Figure 7:**
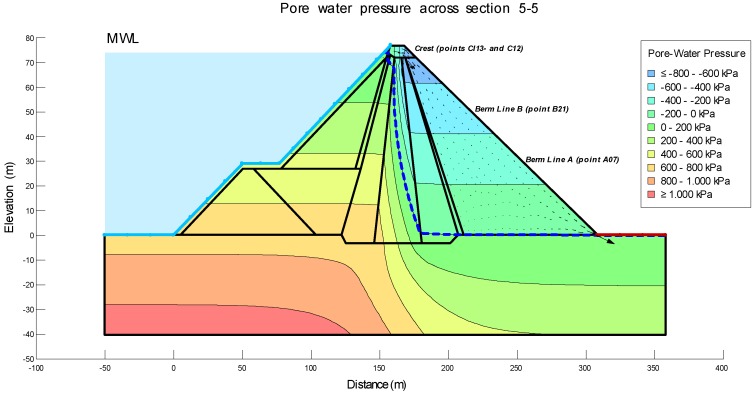
Modeling of the Steady–State Seepage of section 5-5 after the first filling.

**Figure 8:**
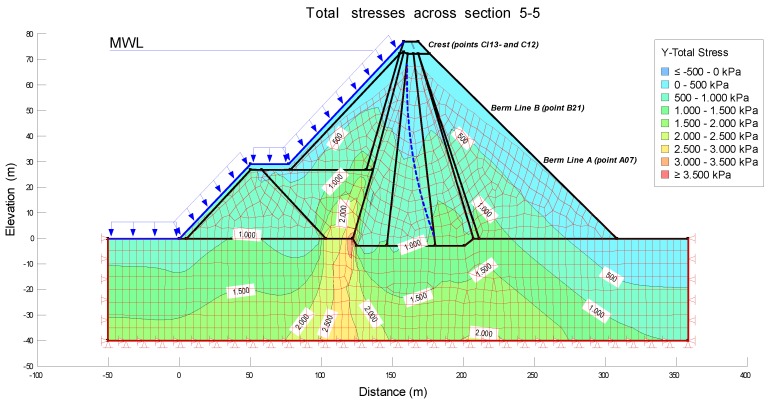
Modeling of Load-deformation of section 5-5 after the first filling.

The level of the reservoir was considered as well. In particular, the water level corresponding to March and July 2008 and March and July 2013 were considered, since in these periods high precision geodetic surveys were carried out. In this way, a continuity of the monitoring will be achieved, and it will be possible to correlate and visualize the results of the displacements. This allows us to obtain the Steady–State Seepage and Load-deformation models that are shown in Figure 7 and Figure 8. 

**Figure 9:**
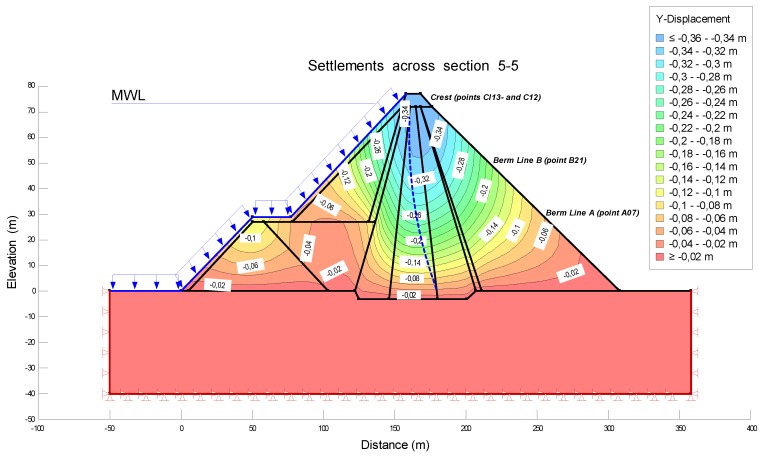
Settlements at the crest and berms across section 5-5 during the period of construction before the first filling.

**Figure 10:**
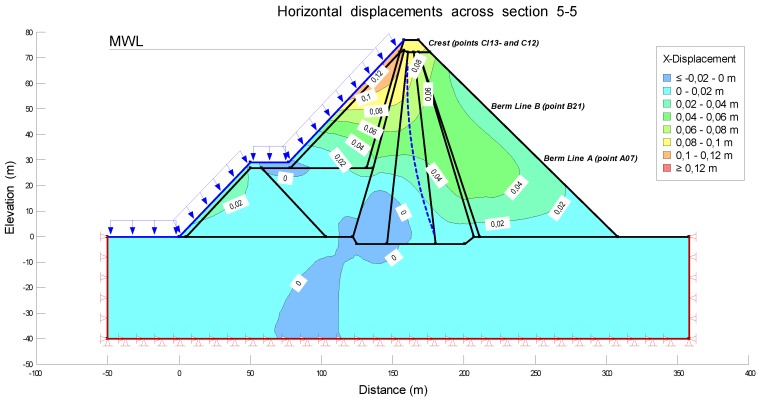
Horizontal displacements at the crest and berms across section 5-5. during the period of construction before the first filling.

Figure 9 and Figure 10 show the deformations (settlements and horizontal displacements) during the period of construction before the first filling. For a better interpretation of the isograms, lines with same displacements are incorporated into the pattern.

The deformations can be visualized in the different areas of section 5-5. The maximum settlement is reached at the crest, with a value of −34 cm. The vertical displacements decrease at the berms, with values equal to −20 cm in line B and −6 cm in line A. The horizontal displacements represent 20% of the settlements, achieving a maximum value of 7 cm at the crest. This percentage agrees with the criteria explained in [21]. According to these, if the settlements are admissible, one can assume that the horizontal displacements are acceptable as well. Therefore, the analysis of the vertical deformations should be sufficient. In the following section, the comparative analysis will focus mainly on the settlements; the horizontal displacements will be analyzed shortly.

The values of the displacements obtained at the crest and the berms agree with those computed at different levels of the nucleus or core. The nucleus can be identified in Figure 4. These values decrease significantly towards lower layers in the frontier of the active power (bedrock). They become insignificant for this type of analysis, but it would be useful if one wanted to compare numerical deformations with possible records of geotechnical instruments installed at the dam.

## 5. Results and Discussion

A comparative analysis between geodetic and numerical displacements is carried out in this section, which includes the construction and operational processes, for the period from 2007 to 2016. The results are shown in Table 4. It is important to remember the construction of the dam lasted from 2004 to 2006. The first control of displacements was carried out in February, April and July 2007, after the first filling of the dam in November 2006. They are considered as geodetic displacements during the construction period, due to no data being available for this period. The displacements estimated by the authors from 2008 to 2016 are considered as the geodetic displacements during the operational period. In particular the geodetic displacements considered in Table 4 for the operational period were those estimated at the control points, A07, B22, C12 and CI13 at line A, B, C and CI, respectively, since they belong to the critical area under study.

The largest differences between the values of the settlements predicted by the numerical method and those obtained by the geodetic measurements can be seen in lines C and B. They occurred during the operational period, with values of −28 cm and −12 cm respectively. They were due to the simplifications and necessary idealizations assumed during the FEM modeling process. In particular, critical sections were considered as homogeneous along their longitude, and the properties of the materials were established according to the general characteristics of an earth fill dam. These characteristics were taken from the normative and similar studies in the country. They are not the actual and proper characteristics of the Arenoso dam. This could also be due to the fact that the geodetic control points were anchored in the superficial layer of the slope when the construction of the dam was finished. Furthermore, the behavior in this area is generally unstable, due to the constant rearrangement of the particles during the consolidation of the soil. Better results were obtained at berm A (next to the base of the slope). The difference decreases, reaching a value of −6 cm. This is due to this area being close to the most resistant stratum (bedrock).

**Figure 11:**
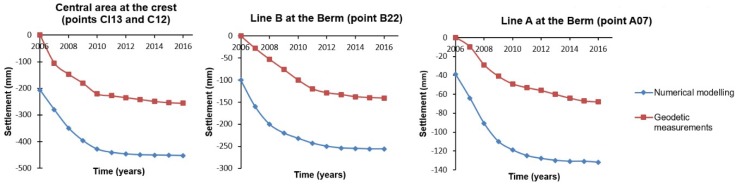
Time series of settlements observed by high precise leveling (red line) and computed by FEM (blue line).

A deeper analysis of the results is carried out from the time series of the control points belonging to section 5-5, in which the displacements reach the maximum value. The most important thing to note from Figure 11 is that, on average, both estimates show a similar trend: the vertical displacements decrease progressively with time, indicating a typical consolidation behavior. Nevertheless, in this process the values of the settlements could increase over time, due the presence of clay (nucleus, drain and filter), which is known as secular settlement. A bias between FEM and geodetic displacements can be seen. This is probably due to the simplifications assumed during the FEM modeling process, and to the geodetic surveys carried out when the construction of the dam was finished.

**Figure 12:**
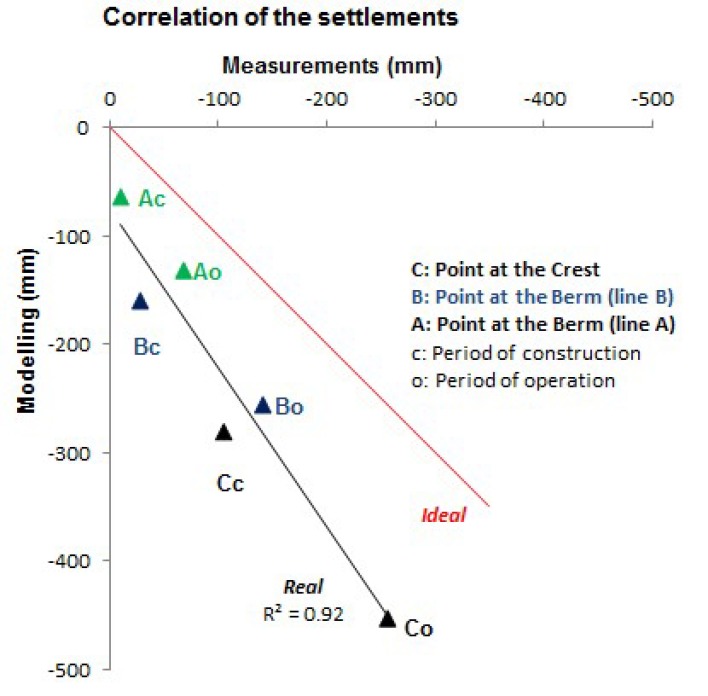
Correlation index between geodetic and FEM settlements.

Figure 12 shows the correlation coefficient between geodetic and numerical settlements computed for the periods of construction and exploitation of the dam. The result suggests a good correlation. The differences with respect to the ideal curve could be due to the high precision leveling surveys begun when the construction period of the dam was finished.

**Figure 13:**
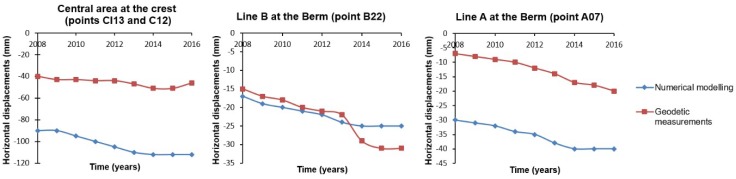
Time series of horizontal displacements observed by GPS measurements (red line) and computed by FEM (blue line).

Finally, we comment briefly on the horizontal displacements computed by GNSS and by FEM. From Figure 13, we can see that the agreement between the horizontal displacements is better than the results obtained for the settlements. A similar pattern in the behavior of horizontal displacements from GNSS and FEM can be seen. The results obtained at line B from 2013 can be considered negligible, since the difference between geodetic and numerical displacements is at error observation level. A bias between FEM and geodetic displacements is presented. The reasons explained above can be used here: the simplifications assumed during the FEM modeling process and the geodetic surveys were carried out when the construction of the dam was finished. In general, from 2013 a trend of attenuation is observed. 

## 6. Conclusions

This study presents an analysis of the deformation monitoring of an earth fill dam. Vertical and horizontal displacements computed using high precision geodetic techniques and FEM modeling have been compared from 2008 to 2016. This comparison reveals a similar pattern between the displacements estimated from geometrical and numerical modeling. In particular, on average the difference between the settlements obtained from high precision leveling observations and numerical modeling during the periods of construction and exploitation is about −15 cm. This result can be due to the simplifications and necessary idealizations assumed during the FEM modeling process. In particular, critical sections were considered as homogeneous along their longitude, and the properties of the materials were established according to general characteristics of an earth fill dam. These characteristics were taken from the normative and similar studies in the country. They are not the actual and proper characteristics of the earth fill dam under study. This could also be due to the geodetic control points being anchored in the superficial layer of the slope when the construction of the dam was finished. The high precision geodetic leveling observations were therefore carried out after the construction period of the dam under study. In this period, and after the first filling of the dam, displacements of this order of magnitude are expected, since earth fill dams deform significantly during this critical phase. The vertical displacements indicate that the main deformation developed faster in the second year after the first filling of the reservoir, and that the magnitude of the displacements is seen to decrease with time, indicating that the dam tends to stabilize. Regarding horizontal displacements, the differences are about 6 cm. In this case, as vertical displacements occur, horizontal movements show a trend towards stability. The results demonstrate the feasibility of using the geodetic method as a pattern for the confirmation and improvement of deterministic modeling methods.

## Figures and Tables

**Table 1:** Horizontal displacements estimated at the external pillars V100, V300 and V500 in mm. Period February–July 2008.

Station	Horizontal Displacement (mm)	Geodetic Azimuth of the Displacement Vector
V100	4.5	65°53′31.8″
V300	10.3	104°33′34.9″
V500	3.9	244°10′43.9″

**Table 2:** Statistics of the semimajor and semiminor axes of the error ellipses at 99% confidence corresponding to the horizontal displacements estimated by GPS. a_min_ minimum value of the semimajor axis; a_mean_ mean value of the semimajor axis; a_max_ maximum value of the semimajor axis; b_min_ minimum value of the semiminor axis; b_mean_ mean value of the semiminor axis; b_mean_ mean value of the semimino axis; n number of points.

Period	a_min_ (mm)	a_mean_ (mm)	a_max_ (mm)	b_min_ (mm)	b_mean_ (mm)	b_max_ (mm)	n
February–July 2008	1.41	2.58	2.83	1.41	2.25	2.83	17
March–July 2013	1.35	1.50	1.95	1.11	1.25	1.59	57
March 2013–August 2014	1.62	1.88	2.40	1.23	1.58	2.01	57
March 2013–September 2015	1.02	1.09	1.05	0.63	0.86	1.14	57
March 2013–September 2016	1.02	1.08	1.26	0.63	0.73	1.14	57

**Table 3:** Technical characteristics of the soil of the dam. *E* is the elastic modulus, *V* is the Poisson ratio, γ is the specific weight, *C* is the cohesion and φ the angle of friction.

Soil (Layer)	Material Type	Material Properties				
		*E* × 10^3^ (kN/m^2^)	*V*	γ (kN/m^3^)	*C* (kN/m^2^)	φ^0^
1	Clay (nucleus)	69	0.30	18.0	50	27
2	Borrow	110	0.30	19.0	-	37
3	Dren Filter	64	0.28	20	-	35
4	Transition	100	0.30	22.0	-	54
5	Riprap	150	0.25	21.0	-	50
6	Conglomerate	1350	0.25	20	15	35
7	Riprap	124	0.24	22.6	-	40
8	Riprap (drainage)	150	0.25	21.0	-	50
9	Coronation (gravel)	200	0.30	20	-	
10	Foundation (bedrock)	8400	0.20	26	-	

**Table 4:** Comparative analysis of geodetic and numerical displacements for the Construction (C) and Operational (O) periods. S stands for settlement and HD for horizontal displacement.

Method	C		O		Lines	
	S	HD	S	HD		
FEM	−0.28	0.09	−0.45	0.11	crest (CI and C)	
	−0.16	0.02	−0.26	0.02	berm B	
	−0.06	0.03	−0.13	0.04	berm A	
Geodetic	−0.06	0.01	−0.22	0.05	crest	CI
	−0.10	0.04	−0.17	0.05		C
	−0.03	0.02	−0.14	0.03	berm B	
	−0.01	0.01	−0.07	0.02	berm A	

## References

[1] Hosbas G., Kartal F., Ersoy N., Erküçük G., Uzel T., Eren K. Surveillance of Oymapinar Dam deformations by means of geodetic control network. Proceedings of the 1st Turkish International Symposium on Deformations.

[2] Barzaghi R., Pinto L., Monaci R. The monitoring of gravity dams: Two tests in Sardinia, Italy. Proceedings of the FIG Working Week (Session TS01F).

[3] Yi T.H., Li H.N., Gu M. (2012). Recent research and applications of GPS-based monitoring technology for high-rise structures. Struct. Control Health Monit..

[4] Grenerczy G., Wegmüller U. (2011). Persistent scatterer interferometry analysis of the embankment failure of a red mud reservoir using ENVISAT ASAR data. Nat. Hazards.

[5] Di Martire D., Iglesias R., Monells D., Centolanza G., Sica S., Ramondini M., Calcaterra D. (2014). Comparison between differential SAR interferometry and ground measurements data in the displacement monitoring of the earth-dam of Conza della Campania (Italy). Remote Sens. Environ..

[6] Milillo P., Perissind D., Salzere J.T., Lundgrenc P., Lacavaa G., Milillof G., Serio C. (2016). Monitoring dam structural health from space: Insights from novel InSAR techniques and multi-parametric modeling applied to the Pertusillo dam Basilicata, Italy. Int. J. Appl. Earth Obs. Geoinf..

[7] Pytharouli S.I., Stiros S.C. (1999). Ladon Dam (Greece), Deformation and Reservoir Level Fluctuations: Evidence for a Causative Relationship from the Spectral Analysis of a Geodetic Monitoring Record. Eng. Struct..

[8] Pytharouli S., Kontogianni V., Psimoulis P., Nickitopoulou A., Stiros S., Skourtis C., Stremmenos F., Kountouris A. (2007). Geodetic monitoring of earthfill and concrete dams in Greece. Int. J. Hydropower Dams.

[9] Pytharouli S.I., Stiros S.C. (2009). Investigation of the parameters controlling the crest settlement of a major earthfill dam based on the threshold correlation analysis. J. Appl. Geod..

[10] Casaca J., Braz N., Conde V. (2014). Combined adjustment of angle and distance measurements in a dam monitoring network. Surv. Rev..

[11] Chrzanowski A., Szostak-Chrzanowski A. Enhancement of deformation modelling in engineering and geosciences by combining deterministic and generalized geometrical analysis. Proceedings of the Annual Conference of the Canadian Society for Civil Engineering and 11th Canadian Hydrotechnical Conference.

[12] Szostak-Chrzanowski A. (2006). Interdisciplinary Approach to Deformation Analysis in Engineering, Mining, and Geosciences Projects by Combining Monitoring Surveys with Deterministic Modeling—Part I.

[13] Vassilis G., Sakellariou M. (2008). Settlemet analysis of the Mornos earth dam (Greece): Evidence from numerical modeling and geodetic monitoring. Eng. Struct..

[14] Yigit C.E., Alcay S., Ceylan A. (2016). Displacement response of a concrete arch dam to seasonal temperature fluctuations and reservoir level rise during the first filling period: Evidence from geodetic data. Geomatics. Nat. Hazards Risk.

[15] De Lacy M.C., Ramos M.I., Gil A.J., Franco O.D., Herrera A.M., Avilés M., Domínguez A., Chica J.C. (2017). Monitoring of vertical deformations by means high-precision geodetic levelling. Test case: The Arenoso dam (South of Spain). J. Appl. Geod..

[16] Romero F., Bobis A., García-Palacios J.J., Cruz D.J. (2007). La Presa del Arenoso. Rev. Obras Públicas.

[17] (2008). LGO 7.0 Online Help Manual. http://leica-geosystems.com/products/total-stations/software/leica-geo-office.

[18] Cocard M., Kahle H.-G., Peter Y., Geiger A., Veis G., Felekis S., Paradissis D., Billiris H. (1999). New constraints on the rapid crustal motion of the Aegean region: Recent results inferred from GPS measurements (1993–1998) across the West Hellenic Arc, Greece. Earth Planet. Sci. Lett..

[19] (2013). User Manual “Stress-Deformation Modeling with SIGMA/W”, An Engineering Methodology.

[20] Spancold (1999). Technical guides of safety dams. Geologic-Geotechnical Studies and Prospecting of Materials.

[21] U.S. Bureau of Reclamation, 2011. Embankment Dams. Design Standards No. 13. Chapter 9: Static Deformation Analysis. http://www.usbr.gov/tsc/techreferences/designstandards/finalds-pdfs/DS13-9.pdf.

